# The effects of slit-pore geometry on capacitive properties: a molecular dynamics study

**DOI:** 10.1038/s41598-020-62943-7

**Published:** 2020-04-16

**Authors:** Morad Biagooi, SeyedEhsan Nedaaee Oskoee

**Affiliations:** 1 Department of Physics, Institute for Advanced Studies in Basic Sciences, P.O. Box 45195-1159 Zanjan, Iran; 2 Research Center for Basic Sciences & Modern Technologies (RBST), Institute for Advanced Studies in Basic Sciences (IASBS), Zanjan, 45137-66731 Iran

**Keywords:** Chemical physics, Porous materials

## Abstract

Ionic-liquids (IL) inside conductive porous media can be used to make electrical energy storage units. Many parameters such as the shape of the pores and the type of IL affect the storage performance. In this work, a simple IL model inside two geometrically different slit-pores is simulated and their capacitive properties are measured. The pores were of finite length, one of them was linear and the other had a convex extra space in the center. The molecular dynamics simulations are done for two, qualitatively, low and high molarities. The pores have been simulated for both initially filled or empty conditions. Differential capacitance, induced charge density, and IL dynamics are calculated for all of the systems.

## Introduction

Supercapacitors have gained much attention for the energy storage^1–4^. They can be made of ionic-liquids (IL) or electrolytes such as water-in-salt between electrodes^5^. ILs are a type of molten salts with melting point below 100 °C^6^. Supercapacitors are also called electrical double-layer capacitors (EDLC) because of the layered configurations of electrolyte or IL near the electrode surfaces. They are expected to have great power performances, high capacitance and theoretically unlimited charge-discharge cycle^7^. However, because of the complexities of these systems, the experiments do not necessarily coincide with the theoretical expectations^8^.

EDLCs are different from chemical batteries. In these systems, there’s no electron transfer between charged particles and the surfaces. If a charged particle or ion gets near to the electrode’s surface, there would be an increase of induced opposite charges on the surface. This is the mechanism of electrical energy storage. EDLC have shorter charge-discharge time because of adsorption-desorption rate of electrolyte’s ions on the electrodes^8^. Since the electric storage in these devices is related to the attraction-repulsion of the ions to the electrode surfaces, the conductive nanoporous media, such as carbide-derived carbon^9^, are a good candidate to be used as electrodes. Rough electrodes possess a bigger amount of surface area and therefore larger capacitance than the smooth ones^10^.

The researches show that charging process in EDLC have a lots of mechanisms and parameters affecting on their capacitive performance, such as self discharge due to redox reactions^11^, overscreening and crowding in dense electrolytes^12^, electrochemical potential windows (EPW) of IL^13^, and changes in volume for the electrodes^14^. Considering all of the known mechanisms at the same time is not possible, especially in experimental^15–18^ and analytical^11^ studies. Because of that, there are different methods invented and used for EDLS simulations such as Molecular dynamics (MD)^19–22^, Monte-Carlo^23,24^, lattice model^7^, density functional theory (DFT)^13,25^, mixed DFT with MD and post-HartreeFock calculation^6^, and machine-learning^26–28^. In addition, modeling and simplifications in simulations gives us some hints about the ongoing microscopic processes in EDLC.

The geometry of the electrodes is an important factor of supercapacitors that highly affects its performance^22^. There are numerous researches on the different electrodes geometries such as, planar electrodes^20,21^, slit-pores^17,23,29^, combining flat and porous electrodes^30^, cylindrical pores^29,31^, spherical electrodes^31^, carbon nanotubes forest^32^, mathematically flat electrodes vs atomic structured electrodes^33^, atomic rough vs atomic non-rough electrode surfaces^10^ and so on.

Equilibrium condition of the pores at zero applied voltage shows another effective factor of the supercapacitors. They are described as ion-philic and ion-phobic pores for filled and empty pores^34^. Ion-phobic pores are expected to have fast charging and higher energy storage than ion-philic ones^23^. Comparing initially filled and empty pores have been done by Kondrat and Kornyshev^35^ with a mean-field model, they have reported that the initially filled pores charging is like diffusive and of the empty pores is a front-like process^8^. Some researches stated that by changing total ion concentration, ion-phobicity of the pores can be controlled, however, this does not seem to be a general rule^23^.

In this work, we focus on the effects of pore geometries. The electrodes geometry chosen for this research is asymmetric, one flat electrode and one slit-pore. In order to do that, two different slit nano-pores are designed and simulated while having IL inside. The simplest symmetric coarse-grained model of ILs is used^20,21^. The electrode walls have no atomic structure and are created of polyhedrons, similar to some other researches^24^. The simulations are done at different molarities with initially filled and empty pores. It is tried to see the effects of the geometry on the capacitive properties. The plan of the paper is as follows; the results are interpreted and discussed in ‘Results and discussion’ section. In section ‘Methods’, the simulation geometry, algorithms, models and parameters are explained.

## Results and discussion

### Neutral thermal configuration

**Figure 1:**
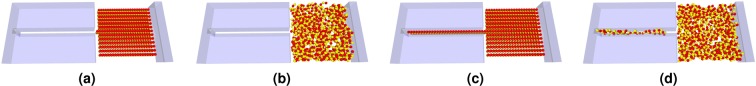
Snapshots of simulation of the IL inside linear slit-pore at 0V applied voltage. (**a**) System No. 2 at *t* = 0. (**b**) System No. 2 at *t* = 10 *ns*. (**c**) System No. 4 at *t* = 0. (**d**) System No. 4 at *t* = 10 ns. This is a comparison between initially empty versus filled pores. In the case of initially empty pore, ions do not penetrate inside the pore while ions remain in the pore when they initially located inside (bottom). The interesting phenomena is existence of an empty region in the entrance of the slit-pore which remains empty during the simulation. (Visualized with Ovito^36^).

**Figure 2:**
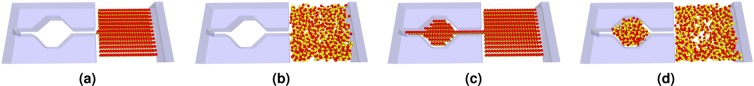
The same situation as Fig. 1 but for convex pore. (**a**) System No. 6 at *t* = 0. (**b**) System No. 6 at *t* = 10 *ns*. (**c**) System No. 8 at *t* = 0. (**d**) System No. 8 at *t* = 10 ns. Again, empty regions are exist at the entrance of slits. (Visualized with Ovito^36^).

Figures 1 and 2 are some snapshots of the system for linear slit-pore and convex pore at zero voltage difference. In both cases, when the pore initially is empty, it remains empty during the simulation and ions do not tend to penetrate inside pores. On the other hand, when pores are initially occupied, ions tend to remain inside the pore except for the entrance of it. In this case, there is an empty region at the entrance of the linear slit-pore where ions do not pass. In the case of the convex pore, ions leave the narrow regions on both sides of the convex pore, they remain outside the pore or occupy the convex and do not leave it during simulation time.

As a simple argument to justify the empty regions, one can refer to the maximum entropy principle. Being in a narrow slit causes ions to be in a pseudo-two-dimensional structure in which ions with opposite charge alternate in each direction and construct a 2D pseudo lattice. This structure imposes an extra order to the IL system and reduces the entropy. Therefore, to maximize the entropy of the system, ions leave the narrow slit to the convex pore or the outside of the electrode. In the case of the linear slit-pore system (Fig. 1), however, after leaving those ions near to the entrance, the remaining ions in the slit construct a connected structure in which the electrostatic force plays the binding interaction role. This binding interaction are strengthened because of the conductive boundary of the slit and makes it hard for ions to leave the slit.

**Figure 3:**
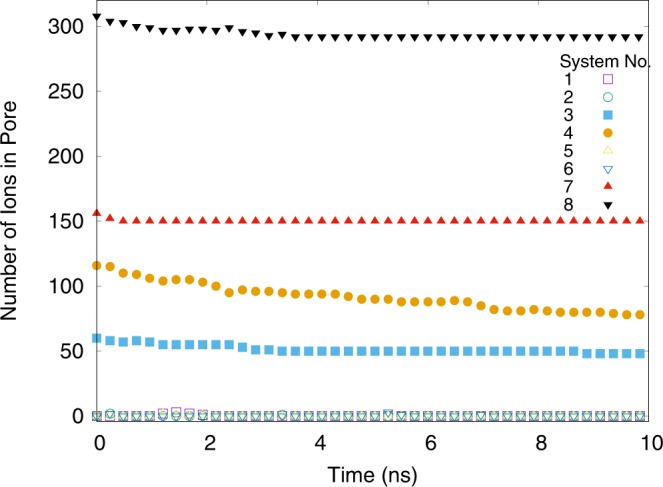
Accumulated ILs inside pore in equilibrium simulation at *V* = 0.0 for different systems. As it is shown, there is only a decreasing number of ions in systems 7 and 8 which can be a result of entropy increase at the pores’ entrance. There are tiny fluctuations around zero for empty systems. (Plotted with Gnuplot^37^).

Figure 3 shows the dynamics of equilibrium runs at zero potential. Systems that are initially filled lose a few particles near pore entrance while the empty ones do not gain new ions, as it has already been mentioned. In systems with initially filled convex pore (Sys. 7 & 8) ions mostly accumulate at the convex hole at the center of pore and as a result, number of ions in pore remains constant with time, except for the early stages of the simulation, where ions at the entrance of pore leave it to the reservoir. In contrast, initially filled linear slit-pores (Sys. 4 & 3), loose their in-pore ions moderately. This is because the system is not at the global maximum of its entropy because of the existence of a pseudo-2D lattice of ions in the slit-pore. Leaving this pseudo lattice and going to the reservoir need to overcome a potential barrier at the entrance of the pore. Thermal fluctuations, however, help ions to leave this pseudo-lattice and therefore, there exists a moderate reduction in the number of ions as a function of time in the linear slit-pores.

A simple justification of the above-mentioned barrier is as follow; an ion belongs to the pseudo-lattice in the pore has to break its links with the other ions to move towards the reservoir. At early stages of the simulation when the whole pore is filled, ions at the entrance of pore have links with both ions in the reservoir as well as pore, therefore they can easily break their links and go to the reservoir. On the other hand, when enough ions leave the pore entrance, an empty space arises, ions on the edge of the pseudo-lattice feel a surface tension due to the lack of symmetry and therefore, it is hard for them to leave the pore.

### IL dynamics

**Figure 4:**
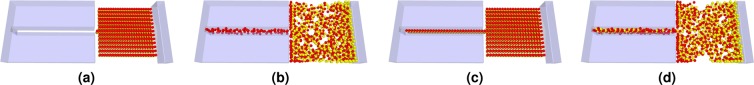
The simulation snapshots of IL inside the linear slit-pore. The electrodes have 4V applied voltage. (**a**) System No. 2 at *t* = 0. (**b**) System No. 2 at *t* = 10 *ns*. (**c**) System No. 4 at *t* = 0. (**d**) System No. 4 at *t* = 10 ns. The yellow spheres are the co-ions for the slit-pores and the red ones are the counter-ions. (Visualized with Ovito^36^).

**Figure 5:**
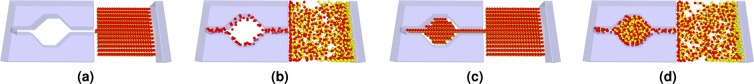
The simulation snapshots of IL inside the convex slit-pore. The electrodes have 4V applied voltage. The yellow spheres are the co-ions for the slit-pores and the red ones are the counter-ions. (**a**) System No. 6 at *t* = 0. (**b**) System No. 6 at *t* = 10 *ns*. (**c**) System No. 8 at *t* = 0. (**d**) System No. 8 at *t* = 10 ns. (Visualized with Ovito^36^).

Figures 4 and 5 are some snapshot of the system of high molarities at finite applied potential difference. The co-ions of the initially filled pores do not leave the pores while empty pores remain empty of co-ions during the charging process. Indeed, there are 3 different ways that an ideal supercapacitor charges: counter-ion adsorption, co-ion desorption, and, ion-exchange. For empty pores, the only method of charging is counter-ion adsorption. For filled pores, however, any of these three methods or any combinations of them can be considered.

**Figure 6:**
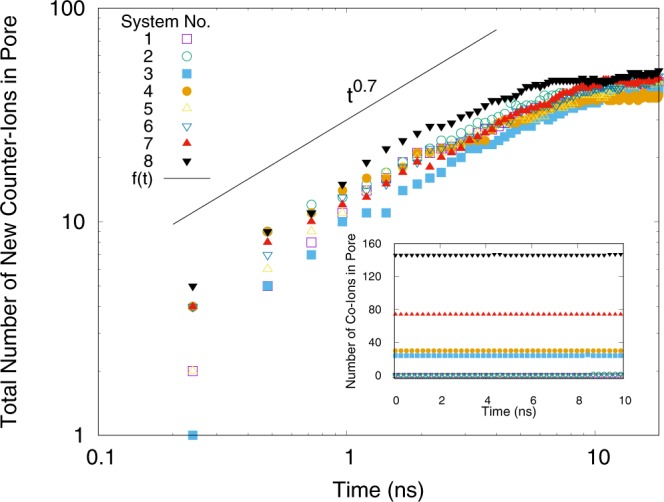
The total number of entering counter-ions into the pore during the charging process versus time at $V\mathrm{=2.0}$ volts. The dynamics of the system are power-law with an exponent less than unity. Inset is the population of co-ions inside the pore as a function of time. This quantity remains constant, which shows that the charging process is just counter-ion adsorption in all different systems. (Plotted with Gnuplot^37^).

To understand the true charging process, we plot the population of the co-ions in the pore as a function of time during the charging process (inset of Fig. 6). Surprisingly, the number of co-ions remains constant, indicating the fact that the only method that contributes to the charging process is counter-ion adsorption. In the other two methods, number of co-ions should decrease due to leaving the pore (co-ion desorption) or due to the exchange with counter-ions (ion-exchange method).

The outset plot of Fig. 6 is the number of entering counter-ions in the pore during the charging process. The plot is in the logarithmic scale. The continuous line is $f(t) \sim {t}^{0.7}$ which is plotted for the sake of clarity. This figure demonstrates power-law dynamics for counter-ions. Although we did not perform a power-law fitting on each individual cures, it is obvious that the power-law exponent is less than unity, indicating on a dynamic slower than the ballistic dynamics which one expects for an ion in an electrical field. This is due to the existence of interactions between ions which cause energy dissipation.

### Capacitive properties

The capacitance for a linear capacitor is defined by $C=q/U$, in which U is the potential difference between the electrodes and *q* is the induced charges. Here *C* is a function of the capacitor geometry as well as the dielectric material inside. Supercapacitors, on the other hand, are more complicated, there are many different parameters such as gravimetric and volumetric capacitance involved in storing the electrical energy. Charge fluctuations play an important role in determining the capacitance of the system and causes the supercapacitor has a nonlinear response to the applied electrical potential^5^. As a measure of this response, one can refer to the differential capacitance (DC) $DC=dq/dU$.

To reduce the finite size effect in our simulation, we use the mean-square fluctuations in the electrode surface charge density to determine the DC;

1 $$DC=\frac{S}{{k}_{B}T}[\langle {\sigma }^{2}\rangle -{\langle \sigma \rangle }^{2}],$$

where $S$ is the electrode internal surface, ${k}_{B}$ is the Boltzmann constant, $T$ is the temperature, and $\sigma$ is the surface charge density. The angle brackets denote ensemble average in fixed applied voltage $U$^38^. Nonlinear dependency of DC to applied voltage $U$ is considered in this relation, since surface charge density is generally potential dependent in our simulations.

**Figure 7:**
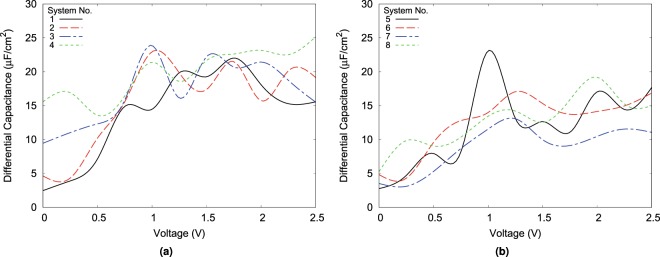
Differential capacitance versus potential for different systems. (**a**) DC for linear slit-pores. (**b**) DC for convex slit-pores. The convex and linear geometries are plotted in different graphs for the sake of clarity. (Plotted with Gnuplot^37^).

The DC plots (Fig. 7) shows a major difference between different configurations of the system. depending on the geometry and initial condition of different systems distinct peaks of the DC appear at different voltages. If these peaks are related to the saddle points in the free energy as it has been mentioned by Merlet *et al*.^38^, it means that free energy is highly affected by details of the initial and boundary conditions of the system.

The DC values are of the order of some of other works^10,23^. Furthermore, it is clear from the Fig. 7 that convex systems typically have a lower differential capacitance value compared to a simple slit-pore. The overall lowest DC is of sys. 8 and the highest is of sys. 4, in which both are filled high molarity systems.

**Figure 8:**
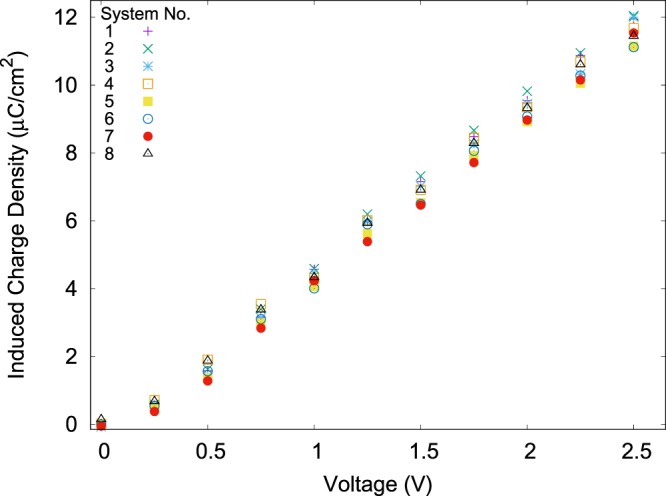
Induced charge density on the electrodes as a function of applied potential. The induced charge value does not show much change for different systems. (Plotted with Gnuplot^37^).

It is reported^23^ that for a slit-pore, the change in molarity does not change induced charge density (ICD) in equilibrium. Figure 8, qualitatively shows the same results for this work. However, one can see a tiny difference (a maximum of 1 μC/cm^2^) for induced charge across systems with different geometries. The lowest ICD are belonged to the convex pores with low molarities (sys. 5 and 7).

## Conclusion

This paper showed how the geometry of two different pores affects their capacitive properties. While results illustrated that differential capacitance follows complicated rules in all systems with different initial and geometric conditions, IL dynamics, and induced charge showed approximately the same behavior in all cases. Furthermore, from three different charging mechanisms, our simulations show that counter-ion adsorption is the only one that contributes. However, more work will be needed for establishing a complete theory of pore geometry effects on the charging process and capacitive properties of the supercapacitors.

### Methods

Details of the simulation method including, simulation setup, the algorithms and the parameters used for this work is discussed in this section. The simulation setup is a combination of ions, pore geometries, and FE meshes. The ions had interactions with each other as well as the conductive boundaries of the porous media. The simulation method was MD, the systems were simulated with the CAVIAR software package, Version 1.0. Details of the simulation procedure are given in the following subsections.

### Ionic-liquid

ILs are modeled as simple spherical symmetric objects with a pure repulsive Lennard-Jones (LJ) potential,

2 $$U(r;\varepsilon ,\sigma )=4\varepsilon \left\{{\left(\frac{\sigma }{r}\right)}^{12}-{\left(\frac{\sigma }{r}\right)}^{6}\right\}-{U}_{cut},\,r{\mathrm{ < 2}}^{\mathrm{(1/6)}}\sigma ,$$

in which, $r$ is the distance between the particles, and the $\varepsilon$ and $\sigma$ are the potential parameters. The cutoff radius is set as ${2}^{\mathrm{(1/6)}}\sigma$ to ensure the repulsive force at any distance, and the ${U}_{cut}$ is set according to $U(r{=2}^{\mathrm{(1/6)}}\sigma ;\varepsilon ,\sigma )=0$ to omit the discontinuity due to the potential truncation at the cutoff distance^20,23,39^. The IL-IL LJ interactions are simulated with ${U}_{IL-IL}(r;{\varepsilon }_{IL},{\sigma }_{IL})$, in which ${\sigma }_{IL}=5$ Å and ${\varepsilon }_{IL}=1\,{\rm{kJ}}/{\rm{mol}}$. The cations/anions have plus/minus one electron absolute charge. Since the target of this study is studying the geometric effects of the electrodes, we can set a similar mass for the cations and anions. As for setting the mass value, there’s a lot of choices as many as the number of known ILs. In addition, the investigation of the different mass values on the results takes some long simulations. It’s good to choose the mass value so that it would not be far from the majority of the experiments and ILs’ applications. A well known IL is BMIM-PF6 that is used in many simulation and experimental studies^20,21,23,33,40,41^. The anion of this IL, PF6 (or Hexafluorophosphate), is one of the most stable anions of ILs and provide the largest EPWs when they are paired with conventional organic cations^13^. It is also one of three widely used non-coordinating anions. So we have choosen the IL mass the same as PF6, i.e. 144*u*.

### Pore geometry

**Figure 9:**
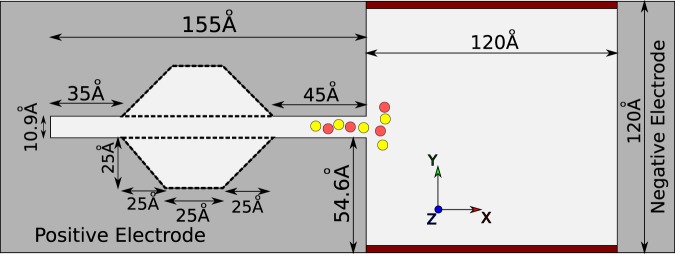
The complete geometry description of the systems. The dashed lines show the extra space of the convex slit-pore compared to the linear one. The geometry is 30Å long along the z-axis, with periodic boundary condition. The reservoir is closed in the y-direction. The electrodes are actually the line interfaces between two light and dark areas. The dark gray areas are forbidden zone for the particles, and are shaded to show the electrodes better. This figure is plotted with Inkscape Version 0.91 (https://inkscape.org/).

Because we want to study the effect of geometry on the capacitance of supercapacitor, two distinct geometries with different electrodes are designed. One of them is a simple slit-pore and the other is a slit-pore with a convex space inside. They are called linear and convex pores in the rest of the paper. Figure 9 illustrates both of the slit-pores geometries in the true scales. This geometry is designed with an open-source computer-aided-design software, SALOME^42^ Version 8.2.0 https://www.salome-platform.org/ and exported as a VTK file format. https://www.vtk.org/VTK/img/file-formats.pdf. The VTK format describes the geometry by triangles.

**Figure 10:**
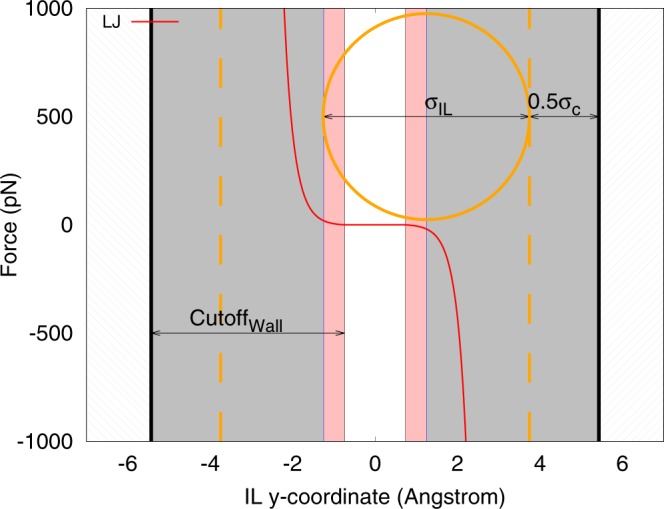
The LJ force, on the IL particles due to the walls of the linear slit-pore, with respect to y-coordinate. The IL particles have a width of 1.475 Å freedom between the walls (white area) in which they have no LJ interaction with the walls. The pink area is the start of the LJ cutoff. The gray area indicates that the distance to the wall is less than the average of ${\sigma }_{IL}$ and ${\sigma }_{c}$. The thick black line shows the position of metallic boundaries. The complete circle shows the size of IL particles compared to the pore width. (Plotted with Gnuplot^37^).

The IL particles interact with the triangles using a discreet-element-method (DEM) algorithm^43^. This algorithm calculates the distance between the particles and the triangles that describe the walls. Using this distance, an interaction potential between ILs and the walls can be set. We have chosen the Eq. 2 for this interaction. In this work, the walls represents the carbon atoms of the pores, meaning ${\sigma }_{c}\mathrm{=3.37}$ Å, and ${\varepsilon }_{c}\mathrm{=1}\,{\rm{kJ}}/{\rm{mol}}$^23^. Using the LJ parameters chosen for IL, the carbon atom parameters, and the Lorentz-Berthelot mixing rules^44^, the parameters for the IL-Wall LJ interactions are calculated as, ${\varepsilon }_{Wall}=\sqrt{{\varepsilon }_{IL}{\varepsilon }_{c}}$ and ${\sigma }_{Wall}\mathrm{=0.5(}{\sigma }_{IL}+{\sigma }_{c})$. Finally, the ${U}_{IL-Wall}(r;{\varepsilon }_{Wall},{\sigma }_{Wall})$ is used to calculate the LJ interaction between the ILs and the pore walls. This force is also purely repulsive, and since the walls are defined stationary, it only acts on the ILs. Figure 10 is an illustrative figure of a slice of the linear slit-pore which demonstrate the different regions of the pore in terms of the interaction of IL particle with pore walls.

### Electrostatic algorithms

Electrodes are conductors, so they can be described by surfaces with constant potential. There are some preliminary efforts to model them as constant-charge surfaces, but the result was not quite realistic^45,46^. Some constant-potential methods were invented for different types of geometries. In addition, some popular methods have been improved for simulating non-flat conductors. As some example of these methods, one can refer to ICC*^47^, as well as an unnamed method introduced by Siepmann *et al*.^48,49^. In this work, the constant potential surfaces are simulated by utilizing the Poisson to Laplace Transformation (PLT) algorithm which is developed by the authors^43,50^. Currently, PLT is only implemented in CAVIAR software package^43^. Instead of defining discrete surface charge density, the PLT method tries to solve the continuous Laplace equation using the superposition principle. This method uses a finite-element (FE) mesh of the pore geometries^51^. Then outer parts of the mesh tags as a surface mesh of different electrodes by which the electric potential difference applies to the system.

To reduce the destructive effect of the finite size, we consider the periodic boundary condition in the *z* direction. As a well-known method of evaluating the long-range electrostatic potential in systems with periodic boundary conditions, one can refer to the Ewald-sum based methods^52–54^. In this work, a 1D Ewald algorithm^55^ for the electrostatic summations is chosen and been used in the PLT algorithm.

### Simulation parameters and tools

There are two slit-pores, each of them has been simulated at different, low and high, IL molarities. In addition, the slit-pore space has been set to be initially filled or empty at the starting point of the simulation (See Figs. 4 and 5). These cases make totally 8 different systems that are summarized in Table 1.

**Table 1:** This table contains the systems initial conditions (I.C.).

No.	Geometry	Pore I.C.	Bulk Mol.	Total Mol.	Qualitative Mol.
1	linear	Empty	1.49	1.34	low
2	linear	Empty	2.30	2.06	high
3	linear	Filled	1.09	1.08	low
4	linear	Filled	2.09	2.08	high
5	convex	Empty	1.49	1.16	low
6	convex	Empty	2.30	1.78	high
7	convex	Filled	1.09	1.08	low
8	convex	Filled	2.09	2.09	high

The system No. is used to refer to them in the results section (Sec. 0). The slit-pore geometry has two types, linear and convex space (see Fig. 9). Pore I.C. is the state of the pore, whether it is filled with ILs or it is empty. Mol. means Molarity in the table.

The systems have been simulated at 11 different, step-like, voltages: $\mathrm{0,0.25,0.5,0.75,1.0,1.25,1.5,1.75,2.0,2.25}$, and, 2.5 volts. There’s a complete symmetry over the charges, so unlike some other works^10,21^, the results won’t change if the applied potential is reversed. The dielectric constant ${\varepsilon }_{r}$ is set to 4.0^23^. Every simulation runs for about 20 ns to reach to equilibrium, then, about $20ns$ extra runs are done for data sampling. Induced charges are sampled every $0.3$ ps. The MD process had a Langevin thermostat at temperature $400K$ with a friction coefficient of $\xi \mathrm{=10}p{s}^{-1}$. The systems are simulated with LJ reduced units^56^. Length is scaled with ion diameter $\hat{x}=\sigma \mathrm{=5}$Å, the mass unit is the mass of ions $\hat{m}\mathrm{=144}$ g/mol, the energy unit is $\hat{\varepsilon }\mathrm{=1}$ kJ/mol, and the unit of charge is one electron. Using the above units to make the governing equation dimensionless conduct us to a time unit equal to $\hat{t}=\sqrt{\hat{m}{\sigma }^{2}/\hat{x}}\mathrm{=6}$ ps. A time-step of $0.001$ in LJ units (6 fs in SI) were used for velocity-verlet integration. Besides, temperature scales as $\hat{T}=\hat{\varepsilon }/{k}_{B}\mathrm{=120.267}$ K, and voltage $\hat{V}=\hat{q}/\hat{\varepsilon }\mathrm{=0.01036}$.

The pore geometries and their mesh are created by SALOME^42^ software. The CAVIAR^43^ software package is used for MD simulations and post-processing the results. The finite-element calculations in CAVIAR are done by using deal.II library^51^. The figures containing IL particles with the geometry are visualized using Ovito^36^ and the line plots are made by Gnuplot^37^.
